# From One of Our Associates

**Published:** 1873-08

**Authors:** 


*From one of our Associates.*

We note the remarks, in your last issue, in regard to medical colleges. It is well that the minds of your readers be frequently drawn to the subject of medical education, as it is one of vital importance to the progress of medical science, and to the dignity and reputation of our noble calling.

The loose manner in which medical colleges, so-called, are, in many instances, organized and conducted; the ignorance of the teachers; the limited and imperfect curriculum of study; the indifference to the primary education and preparation of pupils, and the easy terms of graduation, have greatly lowered the standard and brought disgrace upon the name and dignity of the profession.

Thousands of ignoramuses are thus annually thrown out upon the community, assuming the responsible duties of the physician, occupying the fields of practice, and absorbing the emoluments due to other and better men. As a consequence, quackery is running rife with the country. Men with no merit upon which to build a reputation must resort to deception and fraud to secure business and profit, and having no regard for the honor of the profession, are bringing dishonor and shame upon the noblest of callings. The members of the profession should awake to the great importance of this matter. Let medical men having students advise them not to attend bogus or second class schools of medicine, and let them endeavor to infuse an honorable and elevated tone in the ranks of the profession. ***

THERE can be but little doubt that a change is coming "over the spirit of the dream" of the profession on the intensely vital point of medical education, and the necessity of enforcing the proper educational and *moral* training of students, in order to make accomplished gentlemen and scientific physicians. Our readers may well ponder the words of our friends above—one from Kentucky, and the other of one of the cotton States. There is a remarkable parallelism of thought, showing the outcropping of the professional wants and feelings of the country. We have not the slightest doubt but that, deep down in the hearts of our readers, an amen to these professional appeals well up in responsive tones to the sentiments so beautifully and forcibly uttered by our correspondents. We sincerely wish that every physician within the limits of the Union could read the manly and elevated sentiments of these noble brethren. We hope other friends will send in their thoughts. Let the shout go up from one end of the country to the other, that "unitedly they will enforce the ethics and regulate the educational interests of the profession."

